# Alpha 1-antitrypsin deficiency in patients with chronic obstructive pulmonary disease patients: is systematic screening necessary?

**DOI:** 10.1186/s13104-018-4043-9

**Published:** 2019-01-10

**Authors:** Cláudia Henrique da Costa, Arnaldo José Noronha Filho, Rosa Maria Fernambel Marques e Silva, Thaís Ferrari da Cruz, Valeria de Oliveira Monteiro, Margareth Pio, Rogério Lopes Rufino

**Affiliations:** 1 Chest Department, State University of Rio de Janeiro (UERJ), Av. Marechal Rondon, 381, São Francisco Xavier, Rio de Janeiro, RJ 20950-000 Brazil; 2 Chest Department, State University of Rio de Janeiro (UERJ), Av. Vinte e Oito de Setembro, 77, Vila Isabel, Rio de Janeiro, RJ 20551-030 Brazil

**Keywords:** AAT, COPD, Severe respiratory illness, Immunonephelometry, SERPINA1

## Abstract

**Objective:**

Alpha-1-antitrypsin deficiency is a relatively prevalent, but under-diagnosed, genetic disease. The objective of this study was to assess whether the systematic screening for alpha-1-antitrypsin deficiency in all patients with chronic obstructive pulmonary disease from a tertiary service has an impact on the number of patients being diagnosed with this condition.

**Results:**

Chronic obstructive pulmonary disease patients were screened for alpha-1-antitrypsin deficiency using immunonephelometry. The presence of a mutation was confirmed by molecular study of the SERPINA1 gene or by genetic sequencing, as needed. A total of 551 patients with chronic obstructive pulmonary disease were analyzed. Among these, 40 (7.2%) had some genetic mutation, while 11 (2%) had a Pi*ZZ genotype, resulting in severe respiratory illness. The systematic evaluation of chronic obstructive pulmonary disease patients revealed that screening is an effective method to diagnose alpha-1-antitrypsin deficiency. Early diagnosis may facilitate smoking cessation and initiation of treatment to maintain lung function.

## Introduction

Alpha-1-antitrypsin (AAT) is a glycoprotein produced primarily in hepatocytes and is the main serum protease inhibitor [1, 2]. This enzyme is produced by neutrophils in response to some inflammatory stimuli and aims to destroy aggressive agents. However, since it is one of the few human enzymes capable of degrading elastic fibers, it can lead to the destruction of tissues, especially those of the alveolar walls [3, 4]. Nevertheless, AAT is considered important as it acts as an antagonist of neutrophilic enzymes and is responsible for 90% of all anti-protease activity in human serum; the remaining 10% is undertaken by α_2_-macroglobulin [1, 3, 5, 6]. Serum levels of AAT can be elevated following acute or chronic inflammation, infections, presence of certain neoplasms, severe burns, or during pregnancy and contraceptive use [7].

Although AAT deficiency has long been considered a rare disease, it is now identified as a prevalent genetic disease [8–12], which may have a similar or higher incidence than cystic fibrosis (affecting one in 2000 - 5000 individuals). However, it is considered to be an under-diagnosed disorder [13].

Numerous studies have suggested that the frequency of mutations varies according to the study population. In Europe, the prevalence of the Z deficient allele follows a North–South and East–West gradient, with Scandinavian countries having the highest prevalence of this mutation [14]. The increased prevalence in Northern Europe has been hypothetically attributed to the heightened occurrence of the mutation in the Viking population [15]. It is known that the ZZ genotype is practically absent in Asia and Africa, while the highest prevalence of the S allele is noted in the Iberian Peninsula [1, 14]. The population of Latin America was greatly influenced by migration from Spain, Portugal and Italy, and hence, these mutations may be noted in a sporadic manner in this region [16]. Recently, Blanco et al. developed maps with an inverse distance weighted (IDW)-interpolation method, providing information of Pi*Z distribution worldwide. The authors estimated a total of 91,490 Pi*ZZ in America and Caribbean, and 6162 (95% CI 3053–12,297) in Brazil, however the real scenario is unknown [17]. Brazilian population is under genetic influence from Europeans (specially Portuguese), Africans and Indians, who lived in South America before the colonization.

Deficiency of AAT can result in severe chronic obstructive pulmonary disease (COPD) in young patients, and is a poorly diagnosed condition [18]. In the United States, it is estimated that 2% of all COPD patients are AAT deficient [19]. In general, AAT deficiency should be suspected in patients with COPD with no history of smoking, with a history of early-onset COPD (< 50 years-old), or in those with a family history of AAT deficiency. The presence of emphysema in the lower lobes should also be considered [12, 13, 20]. However, certain guidelines and the World Health Organization suggest that AAT evaluation should be performed in all patients with COPD, regardless of the onset, history of smoking or radiographic imaging pattern [1, 21, 22]. The under-diagnosis of AAT deficiency is a particularly important challenge in primary care, where the largest number of patients with COPD is cared for.

The aim of this study was to evaluate whether systematic screening for AAT increases the number of patients diagnosed with AAT deficiency.

## Main text

**Fig. 1:**
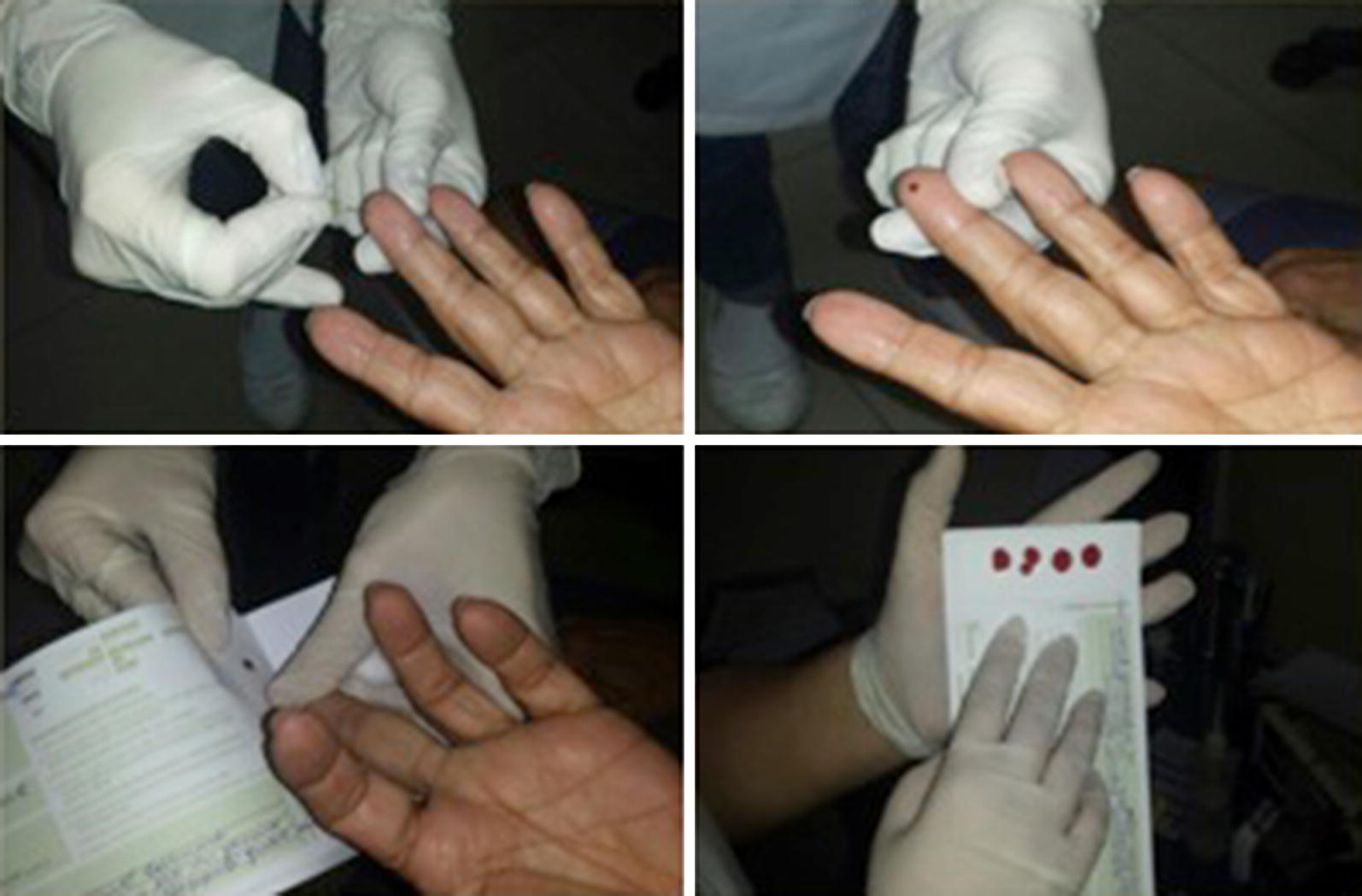
Preparing the dried blood spots. Blood sample was collected by pricking the distal region of a finger and allowing the blood to drop onto a filter paper. The filter paper was put in an envelope and sent to the laboratory

The project was approved by the research ethics committee of the institution (CAAE: 55174616.3.0000.5259). All patients undergoing follow-up at the COPD out-clinic were invited for AAT assessment. Blood sample was collected by pricking the distal region of a finger and allowing the blood to drop onto a filter paper (Fig. 1). The AAT levels were then assessed by immunonephelometry. In patients with AAT levels < 2.64 mg/dL, the presence of a mutation was evaluated by molecular investigation for the presence of SERPINA1 gene or by genetic sequencing, as needed.

Patients with other co-existing disorders (besides COPD) were excluded. The diagnosis of COPD was based on spirometry (forced expiratory volume at first second/forced vital capacity—FEV_1_/FVC < 0.70 in patients with suggestive clinical features). Patients with prior diagnosis of AAT deficiency and family history of proven AAT deficiency were also excluded.

*Respiratory function tests*. All the tests were performed using a spirometer (Vitatrace) coupled to Spiromatic software, and was based on the American Thoracic Society (ATS) 1991 criteria [23]. The predicted theoretical values were those described by Knudson et al. [24].

*Statistical analysis*. The variables were expressed as mean and standard deviation, as the samples showed normal behavior when evaluated by the Shapiro–Wilk test. The statistical package GraphPad 7.0 was used and *p* < 0.05 was considered to express statistically significant values.

## Results

The data of 551 patients, including 283 (51.4%) men and 268 (48.6%) women, were analyzed. Epidemiological and functional data are presented in Table 1. It was observed that 93% patients smoked, but the smoking load was lower in patients with Z homozygosity compared to the total group of patients with COPD, and to all patients with an enzyme deficiency (4 ± 11.2 p-y, 46.5 ± 41.2 p-y, and 34.7 ± 36.9 p-y, respectively); however, the spirometric values were similar to those of other COPD patients (Table 1). There was a tendency for patients with genetic mutation to be younger than the overall group of patients with COPD, but this difference was not statistically significant.

**Table 1:** Demographic and spirometric data from patients included in the study

	All COPD patients n = 551	AAT deficiency n = 40	Pi*ZZ n = 11	p-value
Male: female	283:268	21:19	4:7	–
Age (mean ± SD)	65.2 ± 14.0	63.2 ± 10.2	60.0 ± 7.3	0.19
Smoking (pack-years)	46.5 ± 41.2	34.7 ± 36.9	4.1 ± 11.2	*0.02*
FEV_1_ (% predicted)	50.2 ± 20.6	49.1 ± 20.4	37.7 ± 7.9	0.22
FVC (% predicted)	76.3 ± 22.3	79.4 ± 19.2	78.8 ± 21.4	0.75
FEV_1_/FVC (%)	50.7 ± 13.6	49.0 ± 13.6	41.9 ± 14.6	0.18

The difference among the three groups was analyzed by ANOVA followed by Turkey’s multiple comparison test

*COPD* chronic obstructive pulmonary disease, *SD* standard deviation, FEV1 forced expiratory volume at first second, *FVC* forced vital capacity

**Fig. 2:**
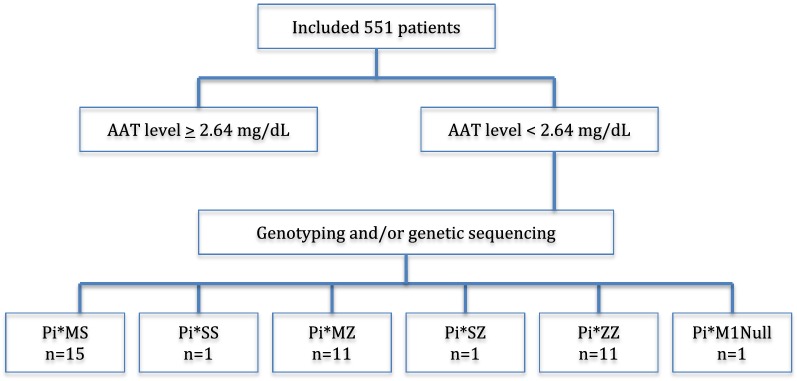
Screening flowchart of patients

About 7.2% patients had some genetic mutation, while 2% (11 of 551 patients) had Pi*ZZ genotype, as presented in the study flowchart (Fig. 2). All patients with Pi*ZZ genotype had the reduced blood levels of this enzyme confirmed. There was no statistical difference between men and women with regard to SERPINE 1 mutation.

## Discussion

The systematic screening for AAT deficiency is feasible and easy to perform. The doctors began to incorporate this without any difficulties in the current study. A considerable increase in the number of patients with COPD diagnosed with genetic mutation of SERPINE 1 and enzyme deficiency was noted during the study period. In 2013, there were 3 patients with the genotype Pi*ZZ while 3 years later, there were 40 patients with genetic mutation, and 11 of them had the Pi*ZZ genotype. The AAT deficiency is an under-diagnosed condition and the difficulty of including this diagnostic evaluation in patients with COPD may be a contributing factor. Thus, the routine request of an AAT dosage for all patients with COPD increases the chances of diagnosing this condition. It is interesting to note that some patients with AAT deficiency were of similar age compared to other COPD patients. In addition, smoking was common throughout the group, including those with the enzyme deficiency. However, the patients with enzyme deficiency had smoked lesser prior to developing the respiratory disease. Of the 40 patients with genetic mutation, 2 were still smokers and discontinued after the mutation was identified. This demonstrates that the knowledge of this characteristic may be a factor that influences smoking cessation.

The AAT replacement was advised only for patients with homozygous Z allele. Currently, of the 11 patients diagnosed with Pi*ZZ genotype, 9 are undergoing the replacement and 2 are awaiting the purchase of the enzyme. Hepatic transaminases and bilirubin levels in patients with severe disease were normal in 10 patients. An increase in transaminases and gama GT was noted in one patient who was an alcoholic, and is being followed-up.

The screening of other family members with a mutation, especially children, can help prevent the onset of respiratory symptoms. Until now, we have been able to evaluate 27 family members (parents, siblings, and children) of patients with AAT deficiency. Among these, 9 individuals with genetic mutation of SERPINA 1 were diagnosed, with Pi*MZ genotype in 5, Pi*ZZ in 2, and Pi*MS and Pi*SZ genotype in 1 individual each. All these individuals are being evaluated for pulmonary and hepatic function. None of the patients with the Pi*ZZ genotype had a history of smoking, but both had respiratory symptoms and one was being treated for asthma. Currently, they are receiving enzyme replacement therapy.

Our data demonstrated that > 7% patients with COPD attending our service had SERPINA 1 mutation, and 11 patients (2% of patients with COPD) had a severe AAT deficiency. Two factors may be contributing to this high percentage of patients with enzyme deficiency. The first is that the study was conducted in a tertiary referral service that treats patients with serious illnesses. Thus, the outcomes may not reflect that of a general group of patients with COPD, since it is based on the percentage of patients with advanced disease, many of whom use supplemental therapy with oxygen. Secondly, the Brazilian population is represented to a large extent by European immigrants, especially Portuguese and Italian, who have a high prevalence of AAT deficiency. Although Z allele is not so prevalent among Portuguese and Italian population, the prevalence of the S allele is high in the Iberian Peninsula [14].

This study did not aim to assess the prevalence of AAT deficiency in the population, but to verify whether the implementation of the routine screening is feasible in public service setting in Brazil. The systematic evaluation of the patients was shown to be effective enough to be recommended as a screening method. Thus, the authors suggest that AAT screening should be performed in all patients with COPD regardless of age, smoking history, gender, or onset of respiratory symptoms. Early diagnosis facilitates smoking cessation and can help initiate changes in order to maintain lung function. In some cases, replacement therapy may be advised.

This study confirms that systematic screening of AAT deficiency among COPD patients increases the chances of diagnosing patients with mutations in the SERPINA1 gene.

This information is important for counseling on smoking cessation as well as for considering replacement therapy in cases of severe AAT deficiency. The methodology used was simple and feasible.

## Limitation

The study was performed in a single center, which receives patients with serious illnesses. Although it is not an unpublished study, this study reinforces the data reported, especially for the region of Latin America, local with little data regarding the AAT deficiency.

## Abbreviations

AAT: alpha-1-antitrypsin
COPD: chronic obstructive pulmonary disease
